# The *International Wound Journal* performance metrics

**DOI:** 10.1111/iwj.14380

**Published:** 2023-09-09

**Authors:** Douglas Queen, Keith Harding

**Affiliations:** ^1^ Medicalhelplines.com Inc; ^2^ Wound Healing Cardiff University

In 2023 the *IWJ* has a lot to celebrate. Firstly, it is our 20th birthday and secondly our performance metrics have continued to improve year on year.

Our impact factor has marginally increased again in 2022. As editors, we are very pleased to confirm that *IWJ*'s impact factor has jumped slightly to 3.1. It has also risen 7 places in the Dermatology category ranking, now 22nd of 70 journals, and 24 places in Surgery, now 49th of 212 journals.

Other important journal metrics also increased in 2022 over 2021 (Table 1) and a glossary of terms is presented in Table 2 to help with understanding of importance of each.

**TABLE 1:** Recent metrics for the *International Wound Journal*.

Metric	2019	2020	2021	2022	% Change
% Self‐citation	12%	16.6%	9.8%	11.8%	2.0%
Article influence	0.605	0.732	0.672	0.700	4.2%
Cited half life	4.7	6.9	5.1	5.6	9.8%
CiteScore	4.7	5.0	5.1	5.6	9.8%
Eigenfactor	0.00558	0.00605	0.00579	0.00548	−5.4%
5‐Year impact factor	2.748	3.629	3.523	3.7	5.0%
Journal citation index	NA	1.19	1.03	1.12	8.7%
Journal impact factor	2.825	3.315	3.099	3.1	0%
SJR	0.877	0.867	0.624	0.693	11.1%
SNIP	1.548	1.592	1.291	1.568	21.5%
Total citable items	187	229	169	269	59.2%
Total citations	3446	4946	5451	6082	11.6%

Abbreviations: SJR, Scientific Journal Rank; SNIP, Source‐Normalised Impact per Paper.

**TABLE 2:** Glossary of terms.

Glossary of metrics
*Self‐citation*—Self‐citation occurs in an article when an author references another of their own publications. This can be a legitimate way to reference earlier findings, but self‐citations can sometimes be unduly made in attempt to inflate an individual's citation count.
*Article influence*—The average influence of a journal's articles over the first 5 years after publication.
*Cited half life*, also known as aggregate cited half life, is a measure sometimes used to evaluate the current interest in a journal.
*CiteScore*—In any given year, the CiteScore of a journal is the number of citations received in that year and in previous 3 years, for documents published in the journal during the total period (4 years), divided by the total number of published documents (articles, reviews, conference papers, book chapters and data papers) in the journal during the same four‐year period.
*Eigenfactor* measures the number of times articles from the journal published in the past 5 years have been cited in the Journal Citation Reports (JCR) year. Like the impact factor, the Eigenfactor score is essentially a ratio of number of citations to total number of articles.
*5‐Year impact factor*—The average number of times articles from the journal published in the past 5 years have been cited in the given Journal Citation Report (JCR) year.
*Journal citation index*—A kind of bibliographic index, an index of citations between publications, allowing the user to easily establish which later documents cite which earlier documents.
*Journal impact factor*—Impact factor is commonly used to evaluate the relative importance of a journal within its field and to measure the frequency with which the ‘average article’ in a journal has been cited in a particular time period. Journal that publishes more review articles will get highest IFs.
*SJR*—The SJR (Scientific Journal Rank) Indicator is a measure of scientific influence of scholarly journals that accounts for both the number of citations received by a journal and the importance or prestige of the journals where such citations come from.
*SNIP*—The SNIP (Source‐Normalised Impact per Paper) is a field‐normalised assessment of journal impact. *SNIP* scores are the ratio of a source's average citation count and ‘citation potential’. Citation potential is measured as the number of citations that a journal would be expected to receive for its subject field. Essentially, the longer the reference list of a citing publication, the lower the value of a citation originating from that publication. *SNIP* therefore allows for direct comparison between fields of research with different publication and citation practices.

While many of the metrics presented in Table 2 represent differing ways of presenting a journal's impact, it is humbling for our editorial and production teams, and our publisher Wiley Inc, to see these increasing metrics across the board. These solidify the *IWJ* as a valuable resource to the scientists and clinicians researching wounds around the world.

A fantastic result, and we thank all involved in this achievement.

As a reader of or a contributor to our journal, you are probably aware of the continued success of the *IWJ*, but here are some updated facts that prove its continued evolution towards the number one international resource for those involved in both the science and practice of wound healing.

To facilitate the continued evolution of the *IWJ*, we have taken some bold steps, in the time periods covered in Table 1, to continue this growth, meeting the continued needs of our readers and authors globally.

1. The biggest step we took was to move to open access, making the *IWJ* available to all readers for free. This was both necessary as the publishing world moves in this direction and our desire to be accessible by all.
2. Tied to the open access of the *IWJ*, it was important to provide mechanisms to those without the resources to pay publishing fees. This is particularly true for some developing countries. Our publisher Wiley has a waiver policy that is applied to the *IWJ* and provides a mechanism for authors in these geographies to publish their work.
3. We increased our editions from 8 to 10 in 2022/2023, and in 2024, we will move to 12 editions. This is necessary as our submission volumes have increased exponentially over the 4 years covered.
4. As this submission backlog increases, it was impacting our submission to publication times. This increase in editions will help reduce this backlog, helping publish important global research in a timely fashion.
5. In 2024, we will be publishing several ‘special’ editions to supplement our annual editions. Watch this space for more information, and you never know you may be approached to be an author in these important initiatives.

All in all, the *IWJ* continues to achieve the international profile and status we desired and is now recognised as the premier international, high‐quality, peer‐reviewed resource in wound care.

As the editorial team, we would like to thank all contributors to this success to date and to also encourage bigger and better things by asking for your continued and increased participation in growing this valuable resource.

